# *In vivo* pharmacokinetic and pharmacodynamic study of co-spray-dried inhalable pirfenidone microparticles in rats

**DOI:** 10.1080/10717544.2022.2149899

**Published:** 2022-11-22

**Authors:** Ji-Hyun Kang, Min-Seok Yang, Dong-Wook Kim, Chun-Woong Park

**Affiliations:** a College of Pharmacy, Chungbuk National University, Cheongju, Republic of Korea; b College of Pharmacy, Wonkwang University, Iksan, Republic of Korea

**Keywords:** Pirfenidone, idiopathic pulmonary fibrosis, dry powder inhaler, co-spray dry, bleomycin

## Abstract

Pirfenidone (PRF) is the first FDA-approved API in the treatment of idiopathic pulmonary fibrosis (IPF). However, PRF induces serious side effects, such as photophobia and gastrointestinal disorder. PRF inhalation can be expected with a lower effective dose and reduced side effects. In this study, PRF was prepared as inhalable co-spray-dried particles for dry powder inhalation. Mannitol, L-leucine (Leu), and NaCl were used as a stabilizer. The kinds and ratios of stabilizers affecting the physicochemical properties of particles were analyzed, including particle size and surface composition, because of the surface enrichment properties of Leu, the most effective stabilizer. The co-spray-dried PRF and Leu microparticle (SD-PL1:1) have the smallest size and highest aerosol performance. The bioavailability was confirmed by *in vivo* pharmacokinetics (PK) studies. In addition, *in vivo* pharmacodynamics (PD) experiments were conducted using a bleomycin-induced IPF rat model. *In vivo* PK experiments demonstrated that pulmonary administration of SD-PL1:1 was 4 times more effective than the oral route. Similar to the PK results, the therapeutic effect was improved when SD-PL1:1 was administered via the pulmonary route compared to the oral route.

## 1. Introduction

Idiopathic pulmonary fibrosis (IPF), the most common type of idiopathic interstitial pneumonia, has an unknown etiology and is unresponsive to treatment. IPF is a chronic lung condition characterized by an aberrant accumulation of fibroblasts and progressive abnormal remodeling of the lung parenchyma with subsequent structural scarring. As a result, IPF promotes inflammatory cell accumulation, the release of cytokines such as TGF-β, and the proliferation and migration of fibroblasts, eventually leading to the accumulation of fibrotic lung tissue (Ley et al., 2014; Rudd et al., 2007). IPF is classified as an orphan disease and occurs at a frequency similar to that of stomach cancer and brain cancer (Hutchinson et al., 2015). The average patient survival is 3–5 years after diagnosis because the disease has a poor prognosis (Hopkinson et al., 2012; Johanson and Sonnenberg, 1990; Schafer et al., 2017). Pharmacotherapy for IPF involves the use of single or combined immunosuppressive agents, such as corticosteroids and azathioprine (Canestaro et al., 2016), or antifibrotic agents, such as pirfenidone and nintedanib (Myllärniemi & Kaarteenaho 2015; Ley et al., 2017; Rogliani et al., 2016). Pirfenidone (PRF) was the first agent to be approved by the Food and Drug Administration (FDA) for the treatment of mild to moderate IPF as an antifibrotic agent. Although the mechanism of action of PRF is unclear, it is known to regulate TGF-β and cytokine levels, which are key factors in IPF progression (Conte et al., 2014), to inhibit fibroblast proliferation, collagen synthesis, and release of collagenases (Hilberg et al., 2012; Macías-Barragán et al., 2010; Schaefer et al., 2011). The oral administration doses of PRF are incredibly high and complex: one capsule or tablet must be administered orally three times daily (600 mg/day of PRF) with food; it is recommended to administer up to three capsules or tablets, three times each day, as needed (1.8 g/day of PRF). High doses often induce various side effects such as gastrointestinal disorders, skin rash due to photo-toxicity, and hepatic dysfunction (Carter, 2011; Taniguchi et al., 2010).

Inhalable dosage forms have been shown to avoid hepatic first-pass metabolism and increase lung distribution, causing low systemic exposure to the drug (Lee et al., 2016; Onoue et al., 2013). Therefore, inhaled administration of PRF can be an appropriate approach to achieve high concentrations locally in target organs and minimize side effects (Seto et al., 2013; Trivedi et al., 2012). Dry powder inhaler formulations of PRF have been developed to reduce its side effects (Onoue et al., 2013; Seto et al., 2016). Dry powder inhalation (DPI) for respiratory delivery has several advantages, such as high stability in the solid-state and comfortable handling. Furthermore, the DPI dosage form significantly decreased the daily treatment dose (Chen et al., 2016; Gradon and Sosnowski, 2014; Lee et al., 2018). The optimal aerodynamic particle size for effective pulmonary delivery to the bronchi-alveoli region is approximately 0.5–5.0 μm (Kang et al., 2021; Park et al., 2013). Inhalable particles are generally prepared by spray drying, a traditional bottom-up method. Spray drying yields dry particles with a narrow particle size distribution as a one-step process in which the sprayed solution or suspension droplet containing the drug dries rapidly. The physicochemical properties of dry particles are determined by the spray-drying parameters and feeding solution formulation (Chen et al., 2016; Kwon et al., 2020; Lee et al., 2016). However, DPI formulations have high surface tension and cohesiveness. To overcome this problem, functional excipients such as polymers, amino acids, and sugar alcohols have been used for spray drying (Ajmera and Scherließ, 2014; Lee et al., 2018; Meenach et al., 2013).

Surface stabilizers are very suitable for enhancing the stability and surface properties of microparticles through co-spray drying. The most common excipients are polymers, amino acids, sugar alcohols, lipids, and inorganic agents (Vehring, 2008). Amino acids, especially leucine, stabilize the surface tension and electrostatic charge by outer coating particles when co-spray drying with a drug (Faghihi et al., 2014; Hasija et al., 2013; Mangal et al., 2015). Leucine is an inhalable dry particle that is rapidly supersaturated on the contraction surface of the droplet during drying (Boraey et al., 2013; Pham et al., 2015). As a result, the prepared inhalable particles exhibit remarkably electrostatic behavior (Barazesh et al., 2018; Muhsin et al., 2016). In addition, using leucine often leads to wrinkled or corrugated surfaces, which reduces cohesion or adhesion forces; therefore, leucine decreases particle size, improving aerosol performance (Chew et al., 2005; Feng et al., 2011; Sou et al., 2013). Sugar alcohols with high Tg values affect the particle formation as hydrogen bond donors. Among the sugar alcohols, mannitol is widely used as a non-reducing sugar alcohol, enhancing the dissolution rate without affecting the particle size distribution and aerosol performance of inhalable particles (Kwon et al., 2020; Lee et al., 2021). Sodium chloride was also used for spray drying as an excipient to reduce particle size and modify the particle shape. The particles were in a stable agglomerated state due to capillary forces and electrostatic interactions affecting cohesion (Chan et al., 1997; Shoyele and Slowey, 2006).

In this study, we prepared PRF inhalable particles for DPI using co-spray drying with different stabilizers. We investigated the effect of stabilizers on the size reduction and aerodynamic performance of PRF particles, using mannitol, L-leucine (Leu), and NaCl as stabilizers. The properties of stabilizers (hydrophilic, hydrophobic, and ionic) that are the most efficient for PRF were investigated. We focused on the types and ratios of stabilizers that affect the physicochemical properties of particles, such as particle size and surface composition. In addition, we performed an *in vitro* aerosol dispersion study of the prepared PRF inhalable particles. Pulmonary delivery of co-spray-dried PRF was evaluated by *in vivo* pharmacokinetic (PK) studies, and pharmacodynamic (PD) studies were conducted using a bleomycin-induced IPF rat model.

## 2. Materials & methods

### 2.1. Materials

Pirfenidone was purchased from Glenmark Pharmaceuticals Ltd. (> 99.9% purity, Bharuch, India). L-leucine (99.0%, Pyeongtaek, Korea) was supplied by Samchun Pure Chemicals Co., Ltd. D-Mannitol (99.0%, Tokyo, Japan) was provided from Tokyo Chemical Industry Co., Ltd. Sodium chloride was purchased from Samchun Pure Chemicals Co., Ltd. (99.0%). Bleomycin sulfate was acquired from the British Pharmacopeia Commission Laboratory (British Pharmacopeia Chemical Reference Substance, Teddington, UK). Water was purified by filtration in the laboratory using Milli-Q (Millipore, Billerica, MA, USA). HPLC-grade solvents were used for the analysis. High-performance liquid chromatography (HPLC)-grade ethanol and acetonitrile were purchased from Honeywell Burdick & Jackson (Muskegon, MI, USA). Sprague–Dawley® (SD) rats were purchased from Samtako Corporation (Osan, Korea).

### 2.2. Methods

#### 2.2.1. Preparation of co-sprayed PRF microparticles with different stabilizers

The co-sprayed PRF microparticles were prepared using a spray dryer (EYELA SD-1000, Rikakikai Co., Ltd., Tokyo, Japan) with sodium chloride (NaCl), L-leucine (Leu), or D-mannitol as a stabilizer at a ratio of 1:1 (w/w). In addition, NaCl and Leu also produced PRF:stabilizer ratios of 1:0.5, 1:0.3, and 1:0.1 (w/w). The total powder concentration of the solution for spray drying was 2% (w/v) in distilled water (DW). The following parameters were used during spray-drying: inlet temperature, 100 °C; outlet temperature, 50 °C; nozzle size, 0.4 mm, feed rate, 10 mL/min; atomization air pressure, 250 kPa; drying air flow rate, 0.35 m^3^/min. PRF microparticles were kept in a glass vial containing silica gel at 4 °C until use.

#### 2.2.2. Physicochemical characterization of co-spray-dried PRF microparticles

The particle size distribution of the raw PRF and prepared microparticles was determined using laser diffraction particle sizing (Mastersizer3000, Malvern Instruments, UK) via the dry dispersion method. The scattering model uses the Mie scattering theory.

The co-spray-dried PRF microparticles were characterized according to their morphology and size distribution. The morphology was examined by scanning electron microscopy (SEM; ZEISS-GEMINI LEO 1530, Zeiss, Germany). The microparticles were spread on carbon tape, and the unattached microparticles were blown off and then platinum coated to a thickness of 200 Å using a Hummer VI sputtering device (Anatech, Sparks, NV, USA). Magnifications were ×1,000, ×5,000, and ×10,000. A voltage of 2 kV was applied.

In addition, XRD patterns of the raw PRF and prepared microparticles were analyzed using a D8 Discover with GADDS (Bruker AXS, Karlsruhe, Germany) at a wavelength of 1.54 Å. The 2θ scans were conducted between 5° and 60°.

Infrared spectroscopy was performed using an FT-IR spectrophotometer (4100 Jasco, Tokyo, Japan). For each spectrum, 16 transient spectra were collected over 650–4000 cm^−1^ for the raw PRF and prepared microparticles.

The thermal properties of the raw material, other excipients, and prepared microparticles were analyzed using a DSC Q2000 (TA Instruments, New Castle, DE, USA) thermal analyzer system. The samples were accurately weighed, loaded into an aluminum pan, and analyzed at a heating rate of 10 °C min^−1^ over a temperature range of 20–350 °C. The thermal response of the prepared sample was calculated using the TA Advantage/Universal Analysis software (v5.2.6).

The true density of the raw material and prepared microparticles were measured using a pycnometer (Pycnometer with thermometer 5 mL, Witeg Labortechnik GmbH, Wertheim, Germany) with hexane, which is an insoluble solvent for all materials. The temperature was maintained at 20 °C. One gram of each sample was used, and all experiments were performed three times.

The surface area and pore volume of the prepared microparticles were measured using an accelerated surface area and porosimetry analyzer (Micromeritics Instrument Corp., Norcross, GA, USA). The samples were degassed at 90 °C for 90 min under nitrogen before analysis, and the surface area was calculated according to the Brunauer–Emmet–Teller (BET) equation. The pore volume between 1.7 and 300.0 nm diameter was calculated according to the Barrett–Joyner–Halenda (BJH) equation.

The surface composition was quantitatively evaluated using X-ray photoelectron spectroscopy (XPS; PHI Quantera II, Physical electronics Inc., Chanhassen, MN, USA). The instrument was equipped with an argon ion gun and monochromatic Al Kα radiation (1486.6 eV) at 25 W (15 kV). The analysis was performed at constant pass energy (PE) of 55 eV (allowing a resolution of ∼0.35 eV) and 280 eV for narrow and survey spectra, respectively. Survey scans were carried out over the 1400–0 eV binding energy range with 1.0 eV steps, and narrow high-resolution scans were run at 0.05 eV. The narrow spectra of C1s (∼283 eV), N1s (∼400.0 eV), O1s (∼530 eV), Na1s (∼1070 eV), and Cl2p (∼197 eV) were quantified using Multipak software (Ulvac-phi, Chanhassen, MN, USA). All raw materials and prepared microparticles were evaluated using XPS to determine the surface composition. Atomic concentrations were calculated using the relative sensitivity factor (RSF). The % composition of PRF on the surface was calculated using a simultaneous equation with the atomic concentration of each raw material (Chow et al., 2017; Mangal et al., 2019; Shetty et al., 2018).

#### 2.2.3. In vitro aerodynamic performance of co-spray-dried PRF microparticles

Based on the USP Chapter 601 specification of aerosols, the aerosol performance of the prepared microparticles was determined using an 8-stage non-viable Andersen cascade impactor with an induction port (ACI, TE-20-800, TISCH Environmental, Inc., Cleves, OH, USA) and RS01® (Plastiape, Osnago, Italy). To prevent particle bounce and re-entrainment, the collection plates of the ACI stage were precoated with silicone oil, and prepared microparticles equivalent to 10 mg of PRF were loaded into a hydroxypropyl methylcellulose hard capsule (HPMC, size 3). Each prepared microparticle was aerosolized with air drawn through it at a controlled flow rate of 60 L/min for 4 s. The number of prepared microparticles remaining in the capsule and deposited onto each collection plate at each stage was measured using HPLC. The HPLC system (Ultimate 3000 series HPLC system, Thermo Scientific, Waltham, MA, USA) was operated at 216 nm with a Luna L11 150 mm × 4.60 mm, 5 μm column (Phenomenex, Torrance, CA, USA). The mobile phase consisting of 70% acetonitrile (v/v) was eluted at a 1.0 mL/min flow rate. The column temperature was maintained at 25 °C, and the volume of each injected sample was 20 μL. The ED and FPF were calculated using the following equations:

$$\begin{array}{c} \text{Emitted}\;\text{dose}\;\left(\text{ED},\;\%\right)=\left(\text{Initial}\;\text{mass}\;\text{in}\;\text{capsule}–\text{final}\;\text{mass}\;\text{remaining}\;\text{in}\;\text{the}\;\text{capsule}\right)/\left(\text{Initial}\;\text{mass}\;\text{in}\;\text{capsule}\right)\times 100\\ \text{Fine}\;\text{particle}\;\text{fraction}\;\left(\text{FPF},\;\%\right)=\left(\text{Mass}\;\text{of}\;\text{the}\;\text{particle}\;\text{in}\;\text{stages}\;2\;\text{through}\;6\right)/\left(\text{Emitted}\;\text{drug}\;\text{amount}\right)\times 100 \end{array}$$

The MMAD and GSD were calculated using information from USP Chapter 601. MMAD was determined from a plot of a mass percentage less than the stated aerodynamic diameter versus the aerodynamic diameter, D50% q, on a log probability paper. The GSD was calculated using the following equation:

$$\text{Geometric}\;\text{standard}\;\text{deviation}\;\left(\text{GSD}\right)=\sqrt{\left(D84.13\%/D15.87\%\right)}$$

#### 2.2.4. In vivo PK studies

The Chungbuk National University Institutional Animal Care and Use Committee approved the experimental protocols and animal care methods used in this study. In the present study, SD rats were kept under specified environmental conditions (temperature: 20 °C, humidity 40% RH) during experiments.

The PK study was performed with 30 mg/kg PRF via the oral or pulmonary route (Onoue et al., 2013; Seto et al., 2016). The oral group was administered PRF solution (10 mg/mL) as 30 mg/kg of PRF via the oral route. The SDH, SDM, and SDL groups were administered SD-PL 1:1 as 30, 15, and 7.5 mg/kg of PRF using an insufflator (DP-4, Penn-Century Inc., Philadelphia, PA, USA), respectively. Blood was collected via the orbital vein at 10, 20, 30, 60, and 180 min after administration (*n* = 6). Plasma was immediately separated from the blood samples via centrifugation at 1,000 g for 2 min. After finishing the administration for each group, four rats were sacrificed at each of the following time points: 10, 30, 60, and 180 min. Lung tissues were placed in 10 mL of saline and homogenized at 12,000 g for 3 min using a homogenizer. Acetonitrile (ACN) was added to each plasma and lung tissue sample in a 1:1 (v/v) ratio to precipitate the protein, and the sample solutions were centrifuged at 12,000 g for 5 min to obtain a supernatant. All samples were analyzed using LC/MS/MS to quantify the PRF levels. All analyses were performed using an Agilent 1260 Series liquid chromatograph (Agilent, Waldbronn, Germany) and a 3200 Q TRAP LC/MS/MS system (AB Sciex, Framingham, MA, USA) equipped with an electrospray ion source (Turbo V^TM^ Ion spray, AB Sciex). The column (2.1 mm × 50 mm, 5 μm particle-size; Waters Xterra MS C18, Waters Copr., Milford, MA, USA) was used with a guard column. The mobile phase was a linear gradient from 95% A (0.1% formic acid in water)/5% B (0.1% formic acid in acetonitrile) to 5% A/95% B. The flow rate was 0.4 mL/min. The determination of PRF and carbamazepine as an internal standard was performed by LC/MS/MS operated in multiple reaction monitoring (MRM) mode (m/z 186.2 → 65.1 for pirfenidone and m/z 237 → 194 for internal standard) and positive ion electrospray ionization interface.

#### 2.2.5. In vivo PD studies

IPF was induced through a single administration of 50 µL of BLM solution (5 mg mL-1) via intratracheal instillation (ITI) (Comeglio et al., 2017; Comeglio et al., 2019; Della Latta et al., 2015; Kandhare et al., 2015; Marudamuthu et al., 2019). The negative control (NC) group did not receive BLM, and the positive control (PC) group did not receive drugs after administering BLM. One week after BLM administration, four PRF administration groups (the oral, SDL, SDM, and SDH groups) were established, similar to those used in the PK study. The oral group was administered PRF solution (10 mg/mL) as 30 mg/kg of PRF via the oral route. The SDH, SDM, and SDL groups were administered SD-PL 1:1 as 30, 15, and 7.5 mg/kg of PRF using an insufflator (DP-4, Penn-Century Inc., Philadelphia, PA, USA), respectively. Each of the six groups comprised 10 animals.

The day after the last drug administration, lung function was evaluated using double-chamber plethysmography (DCP, Emka Technologies, Paris, France) (Lee et al., 2021). Blood and lungs were harvested by sacrificing six animals in each group. Blood was collected in an EDTA tube and gently shaken. Plasma was immediately separated from the blood samples by centrifugation at 1,000 g for 2 min. Bronchoalveolar lavage fluid (BALF) samples were obtained by injecting 3 mL PBS into the lungs of the rats. Interleukin 6 (IL-6), tumor necrosis factor-alpha (TNF-α), transforming growth factor-beta (TGF-β), matrix metalloproteinase-2 (MMP-2), total collagen, and hydroxyproline levels in BALF were quantified using ELISA kits (Comeglio et al., 2017; Della Latta et al., 2015; Kandhare et al., 2015; Li et al., 2015; Marudamuthu et al., 2019; Wu et al., 2016). The lungs were dissected and fixed with 4% paraformaldehyde for morphological analysis of the pulmonary vessels. Paraffin-embedded sections were processed for hematoxylin–eosin (H&E) staining and Masson’s trichrome staining for optical microscopy. Each stained lung slice was magnified 20 times to obtain five microscopic images at random locations per sample. Ten researchers, who were previously trained on the modified Ashcroft score standard, observed each blinded microscope image and scored according to the modified Ashcroft score standard (Hübner et al., 2008; Kim et al., 2021; Mahmutovic Persson et al., 2020; Park et al., 2021).

#### 2.2.6. Statistical analysis

Statistically significant differences were evaluated using one-way analysis of variance (ANOVA) with LSD or Games-Howell *post hoc* test using SPSS version 23 (SPSS Inc., Chicago, IL, USA). Statistical significance was set at *p* < 0.05. In addition, the difference between the two groups was determined using Student’s *t*-test with significance set at *p <* 0.05.

## 3. Results and discussion

### 3.1. Preparation and characterization of co-spray-dried microparticles

The particle size of the raw PRF was too large to inhale at a Dv50 of 127 μm. When spray-dried with PRF alone, the Dv50 for particle size was 54.3 μm, implying that the particle size was smaller than that of raw PRF but still not inhalable (SD-PRF in Table 1). Owing to the strong, cohesive force of PRF, it aggregates and has a large particle size even if it is sprayed and dried with a small droplet.

**Table 1.** Particle size distribution of co-spray-dried PRF microparticles.

	Raw-PRF	SD-PRF	SD-PM 1:1	SD-PN 1:1	SD-PN 1:0.5	SD-PN 1:0.3	SD-PN 1:0.1	SD-PL 1:1	SD-PL 1:0.5	SD-PL 1:0.3	SD-PL 1:0.1
Stabilizer	–	–	Mannitol	NaCl	NaCl	NaCl	NaCl	Leucine	Leucine	Leucine	Leucine
PRF: stabilizer	–	–	1:1	1:1	1:0.5	1:0.3	1:0.1	1:1	1:0.5	1:0.3	1:0.1
Dv10 (μm)	41.7	19.2	20.7	4.64	8.96	11.2	18.3	0.902	2.35	1.90	3.80
Dv50 (μm)	127	54.3	64.2	11.4	21.3	28.7	62.8	4.28	6.44	5.18	8.14
Dv90 (μm)	256	161	330	104	92.7	149	273	9.73	18.7	10.8	16.3
Span	1.68	2.607	4.81	8.73	3.92	4.81	4.05	2.06	2.54	1.72	1.54

SD-PRF, spray-dried PRF; SD-PL, co-spray-dried PRF with L-leucine; SD-PN, co-spray-dried PRF with NaCl; SD-PM, co-spray-dried PRF with mannitol.

**Figure 1.**
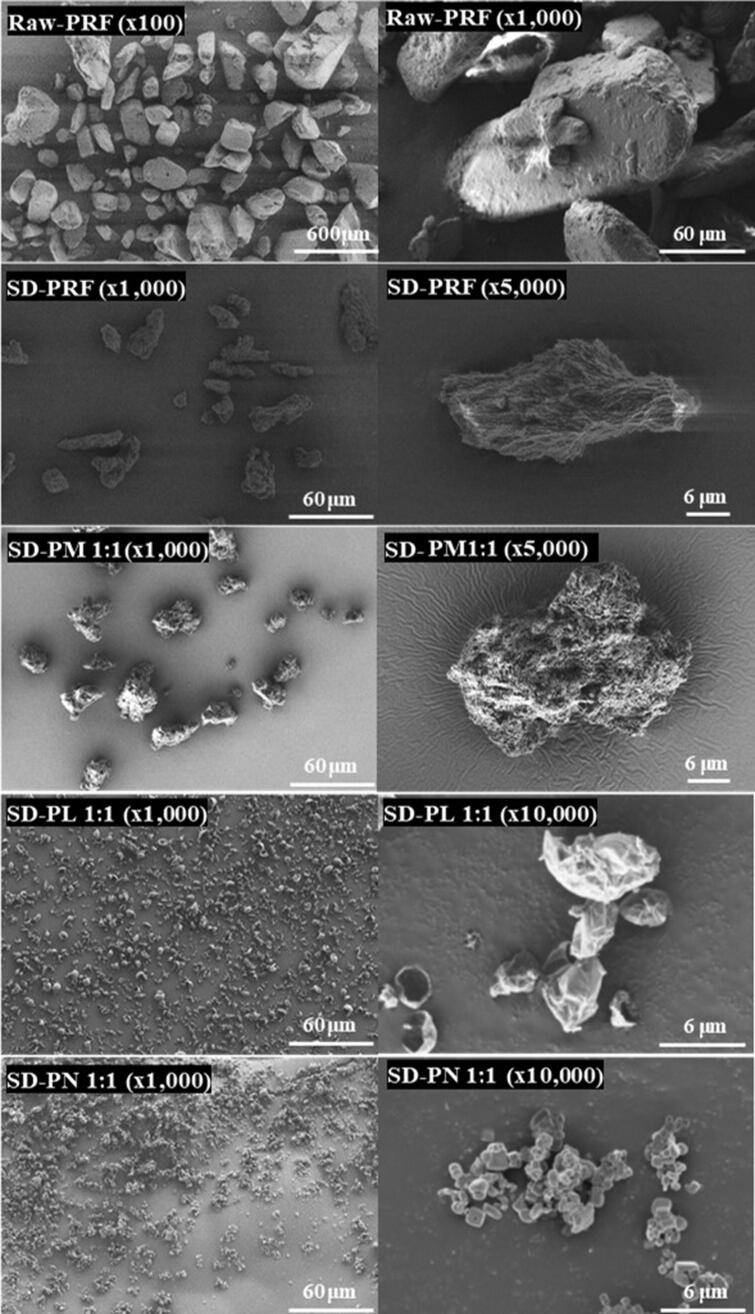
Scanning electron microscopic image of PRF and co-spray-dried microparticles. SD-PRF, spray-dried PRF; SD-PL, co-spray-dried PRF with L-leucine; SD-PN, co-spray-dried PRF with NaCl; SD-PM, co-spray-dried PRF with mannitol.

To improve these phenomena, particles were prepared with PRF and stabilizers such as mannitol, NaCl, and Leu using co-spray dry methods. SD-PM 1:1, SD-PN 1:1, and SD-PN 1:1 have D_V_50 values of 64.2 μm, 11.4 μm, and 4.28 μm, respectively. With a PRF: stabilizer ratio of 1:1, co-spraying with mannitol did not show a significant difference from SD-PRF; instead, the Dv90 was larger than that of raw PRF. Co-spraying with NaCl and Leu, in contrast, successfully produced small particles, as observed in the SEM image in Figure 1. Compared to raw PRF, the sizes of SD-PRF and SD-PM 1: 1 were smaller. However, the particles were larger than SD-PL 1:1 and SD-PN 1:1. SD-PL 1:1 has a wrinkled shape closer to a sphere than other microparticles and has a particle size of approximately 6 μm. SD-PN 1:1 is a mass of small particles on a smooth surface with a cubic shape, whose presence has been attributed to the shape of NaCl crystals. Although the particle size distribution results by diffraction analysis were significant, MMAD may appear smaller in aerodynamic performance studies. Therefore, except for mannitol, the ratios of NaCl and Leu were reduced to confirm this tendency.

**Figure 2.**
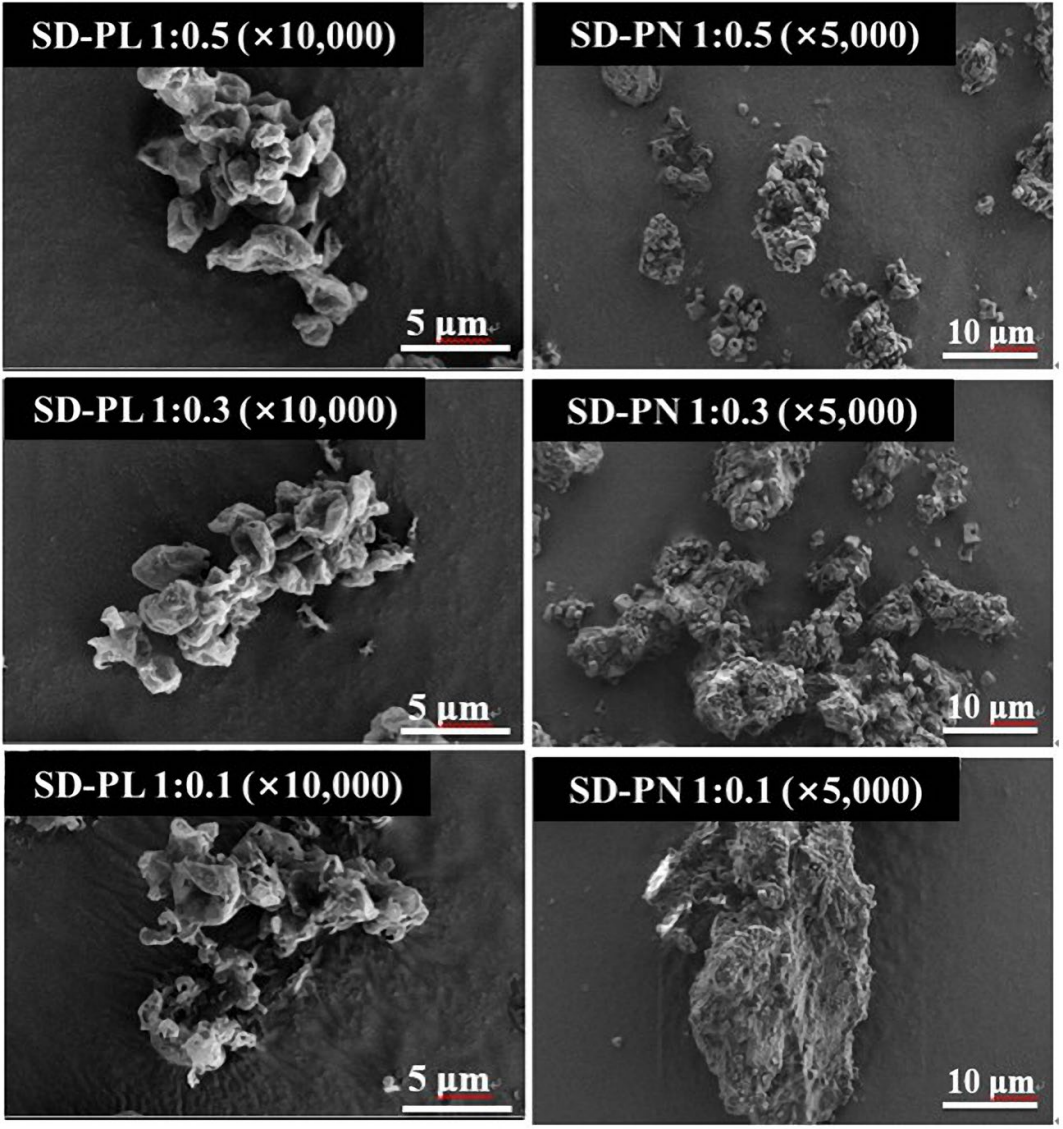
Scanning electron microscopic image of SD-PL and SD-PN particles. SD-PL: co-spray-dried PRF with L-leucine; SD-PN: co-spray-dried PRF with NaCl.

Both NaCl and Leu increased the particle size as the ratio decreased. Therefore, the size of SD-PL 1:0.1 is smaller than that of SD-PN 1:1, and Leu is expectedly the most suitable stabilizer (Table 1 and Figure 2). In the case of SD-PN, a cubic shape (presumed to be NaCl particles) was embedded in the PRF particles. Based on this, it is expected that SD-PN 1:1 is linked to PRF particles rather than simply a cohesive attachment of NaCl particles. In the particle size distribution (PSD) results of SD-PL, the larger the proportion of PRF, the larger the number of SD-PL particles. However, in the SEM image, at all ratios of SD-PL, particles with the same size and shape as SD-PL 1:1 aggregated to form a large agglomerate. This is because the proportion of the stabilizer is low, and the proportion of PRF on the surface is relatively high; thus, the particles are bundled by the strong, cohesive force of the PRF. Consequently, mass particles can pass through the grid structure within the DPI device and disperse. Therefore, it was considered more appropriate to determine MMAD through *in vitro* aerodynamic performance studies. Based on the particle size distribution and SEM images, Leu or NaCl is more efficient as a surface stabilizer for reducing the size of PRF particles in the co-spray dry process than mannitol.

**Figure 3.**
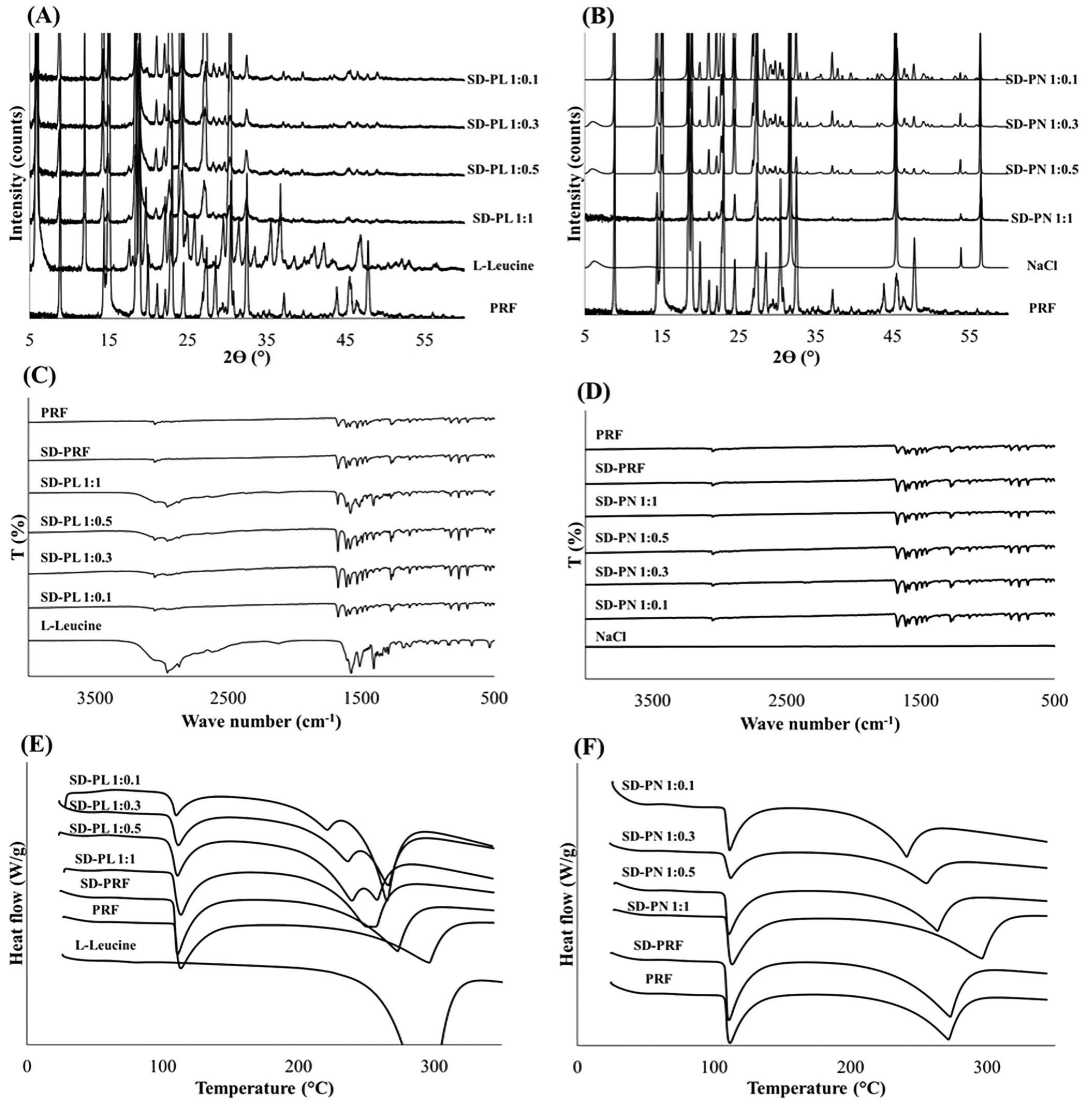
Physicochemical properties of SD-PL and SD-PN (A) X-ray diffraction (XRD) pattern of SD-PL and (B) SD-PN. (C) Fourier-transform infrared (FT-IR) spectra of SD-PL and (D) SD-PN. (E) Differential scanning calorimetry (DSC) thermogram of SD-PL and (F) SD-PN. SD-PL: co-spray-dried PRF with L-leucine; SD-PN: co-spray-dried PRF with NaCl.

Figures 3 (A) and (B) shows the XRD patterns for each SD-PL and SD-PN. In both SD-PL and SD-PN, PRF was in a crystalline form, and each stabilizer seemed to maintain crystallinity. No crystalline change was observed due to the interaction between PRF and the stabilizer. This is also shown by the FT-IR spectra in Figures 3 (C) and (D). There were no peak shifts or new peaks for the PRFs and stabilizers. Therefore, each PRF and stabilizer maintained its crystallinity and was distributed in the particle. Figures 3 (E) and (F) show the DSC thermograms. In Figure 3 (E), the peak around 110 °C, which is the melting point of PRF, is observed in the SD-PL. Thus, it can be seen that PRF exists inside the SD-PL in a crystalline form. It was discovered that SD-PL overlapped with the melting peak of Leu and the boiling peak of PRF at approximately 300 °C because Leu is affected by the melted liquid PRF, so the melting of Leu is also shifted to lower temperatures. However, this interaction generally occurs outside the temperature range in which the drug is used. Therefore, SD-PL particles are considered to be thermodynamically stable because there is no significant change in PRF melting peak at 110 °C. In Figure 3 (F), SD-PN particles also have PRF melting peaks near 110 °C, so PRF inside SD-PN particles also exists in crystalline form. In addition, as a shift in the melting point of PRF is not found, SD-PN particles are also considered thermodynamically stable. Confirmation of the physicochemical properties across various aspects implies that the distributed SD-PL and SD-PN particles maintain their respective crystal forms, and there is no special interaction.

Table 2 shows the evaluation results for the true density and surface characteristics. The true densities were 1.688 and 0.544 g/mL for SD-PN and SD-PL, respectively. The density of NaCl added as a stabilizer to SD-PN 1:1 was measured to be approximately 2.1 g/mL (data not shown). Because of the high density of NaCl, the density of SD-PN 1:1 was also high. In contrast, Leu had a density of about 1.1 g/mL, and the density of SD-PL 1:1 is lower than this, owing to the high diffusion coefficient of Leu in the water. Leu rapidly diffuses onto the surface of the droplets during the spray-drying process, followed by crystallization and drying (Alhajj et al., 2021; Wang et al., 2021). As there are many Leu on the surface of the particles, the particles have low density. Owing to the surface enrichment properties of Leu, the particle structures are corrugated or folded after spray drying (Kaewjan & Srichana; Vehring, 2008; Wang et al., 2021). The surface area and total pore volume of SD-PL 1:1 were also higher than those of SD-PN 1:1 due to the presence of wrinkles and a folded shape (Table 2). These properties are aerodynamically advantageous and may effectively increase inhalation efficiency. In addition, the surface features of Leu can be identified by the percentage of PRF present on the surface via XPS. For SD-PL 1:1, the ratio of PRF to the surface was less than approximately 1%. Only Leu was present on the surface of the particles. Thus, owing to the surface enrichment of Leu, co-spraying Leu and PRF obtained the smallest particles compared to other stabilizers. The proportion of PRF on the surface is the lowest, reducing the cohesive force and the co-spray-dried microparticles. As a result, Leu is considered the most efficient stabilizer yielding inhalable microparticles, and SD-PL 1:1 is expected to be the most advantageous for inhalation based on particle size and surface characteristics.

**Table 2.** True density and surface characteristics of co-spray-dried PRF microparticle (*n* = 3, mean ± standard deviation).

	True density (g/mL)	Surface area (m^2^/g)	Total pore volume (μL/g)	surface PRF (%)
SD-PN 1:1	1.688 ± 0.044	0.5199 ± 0.001	1.615	25.97 ± 2.55
SD-PL 1:1	0.544 ± 0.061	1.0539 ± 0.009	4.433	0.99 ± 0.01

SD-PL, co-spray-dried PRF with L-leucine; SD-PN, co-spray-dried PRF with NaCl.

### 3.2. In vitro aerodynamic performance of co-spray-dried microparticles

**Figure 4.**
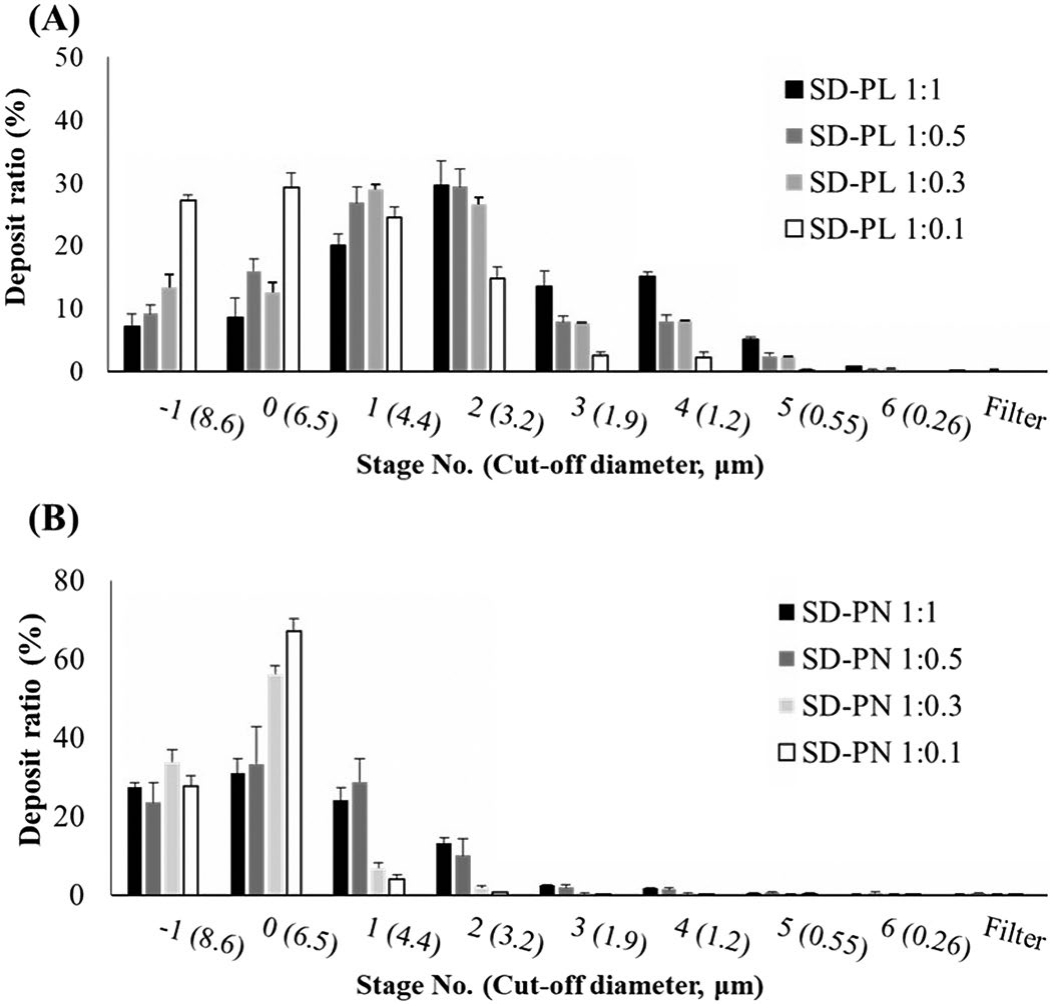
*In vitro* aerosol performance of SD-PL and SD-PN particles (*n* = 3, mean ± standard deviation). (A) SD-PL and (B) SD-PN particles. SD-PL: co-spray-dried PRF with L-leucine; SD-PN: co-spray-dried PRF with NaCl.

Figure 4 shows the deposit ratios at each stage of the ACI for SD-PL and SD-PN particles. SD-PL particles were generally deposited in stages 2 and above, whereas SD-PN particles were mainly deposited in stages −1 and 0. SD-PLs have high inhalation efficiency because they had deposited at stage 1 or higher stage representing deep lungs. The lower the ratio of both stabilizers, the greater the percentage of deposits in the lower stage, and this trend is similar to that of the PSD results. As shown in Tables 3 and 4, among the microparticles using Leu as a stabilizer, SD-PL 1:1 had the highest FPF (33.69%), indicating the highest inhalation efficiency. MMAD was also the lowest at 3.85 μm; in contrast, although the ratio for SD-PN 1:1 particles was the highest among the SD-PN particles using NaCl as a stabilizer, the FPF was very low at 11.37%, and the MMAD was 6.96 μm, which is too large to deposit in the deep lung. SD-PN 1:1 particles were expected to break and reach deep lungs when inhaled because microscopic particles appeared to be agglomerated in the SEM image. The observations noted differed from expectations, and the FPF was very low. As indicated in Table 2, PRF in SD-PN 1:1 acts as a bridge between cubic particles presumed to be NaCl in the SEM image. It is a large particle that does not agglomerate small particles, and as it exists as a large particle, it was deposited in the low stage. Furthermore, SD-PN 1:1 has a high density and is considered disadvantageous for aerosolization. On the contrary, SD-PL particles using Leu have features such as low true density, large surface area, wrinkles, and small particle size; thus, it is considered that aerosolization is improved, and the particles would reach deep lungs. Therefore, in subsequent PK and PD studies, SD-PL 1:1 was administered via the pulmonary route to proceed with the evaluation.

**Table 3.** Aerosol performance characteristics of SD-PL and SD-PN particles (*n* = 3, mean ± standard deviation).

	MMAD ± SD (μm)	GSD ± SD	ED ± SD (%)	FPF ± SD (%)
SD-PL1:1	3.85 ± 0.21	1.46 ± 0.08	98.68 ± 0.11	33.69 ± 1.19
SD-PL1:0.5	4.55 ± 0.25	1.60 ± 0.20	98.94 ± 0.19	30.87 ± 3.19
SD-PL1:0.3	4.68 ± 0.09	1.66 ± 0.04	99.07 ± 0.31	15.88 ± 0.49
SD-PL1:0.1	6.86 ± 0.16	1.44 ± 0.04	99.09 ± 0.07	8.00 ± 1.25
SD-PN1:1	6.96 ± 0.24	1.42 ± 0.06	97.96 ± 0.37	11.37 ± 0.26
SD-PN1:0.5	6.80 ± 0.56	1.39 ± 0.12	97.78 ± 0.46	6.76 ± 1.63
SD-PN1:0.3	8.03 ± 0.12	1.18 ± 0.01	98.57 ± 0.09	2.81 ± 0.20
SD-PN1:0.1	7.98 ± 0.05	1.14 ± 0.01	98.76 ± 0.70	2.56 ± 0.50

**Table 4.** Pharmacokinetic parameters of pirfenidone (PRF) in SD rats (*n* = 6, mean ± standard error).

	Oral	SDL	SDM	SDH
C_max, plasma_ (μg/mL)	10.7 ± 0.53	3.7 ± 0.28*	21.8 ± 2.14^**,##^	35.4 ± 3.02^††^
AUC_inf, plasma_ (hr μg/mL)	8.6 ± 1.13	3.8 ± 0.38	15.0 ± 2.00*^,##^	33.4 ± 2.73^††^
Relative bioavailability (%)	–	175.4 ± 17.60	351.4 ± 46.79^##^	390.5 ± 31.84^##^
C_max, lung_ (μg/g)	6.8 ± 0.02	3.0 ± 0.29**	5.7 ± 0.22^##^	18.7 ± 0.83^##,††^
AUC_inf, lung_ (hr μg/g)	3.6 ± 0.32	1.2 ± 0.05*	12.1 ± 0.88^**,##^	10.6 ± 0.19^**,##^
Relative lung distribution (%)	–	138.6 ± 5.20	679.8 ± 49.65^##^	298.1 ± 5.30^†^

* ANOVA, *p*-value < 0.05 compared with oral; ** ANOVA, *p*-value < 0.005 compared with oral; ## ANOVA, *p*-value < 0.005 compared with SDL; † ANOVA, *p*-value < 0.05 compared with SDM; †† ANOVA, *p*-value < 0.005 compared with SDM.

### 3.3. In vivo PK studies

**Figure 5.**
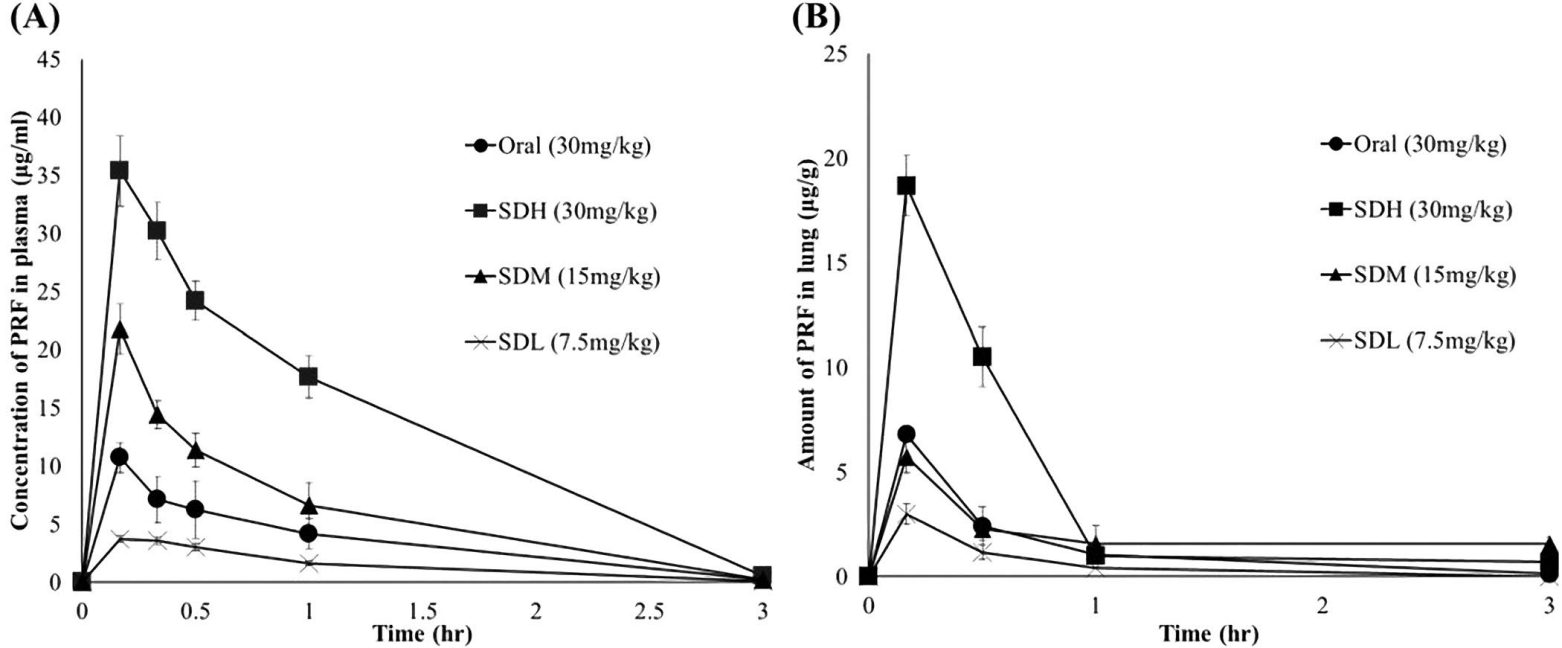
Plasma concentration and lung deposition amount of pirfenidone (PRF) in SD rats (*n* = 6, mean ± standard error). (A) Mean plasma concentration versus time curves of PRF after 30 mg/kg PRF administration to SD rats. (B) Lung deposition amount of PRF after 30 mg/kg PRF administration to SD rats. Oral: PRF solution administered via oral route as 30 mg/kg of PRF; SDH: SD-PL 1:1 administered via the lungs as 30 mg/kg of PRF; SDM: SD-PL 1:1 administered via the lungs as 15 mg/kg of PRF; SDL: SD-PL 1:1 administered via the lungs as 30 mg/kg of PRF.

Figure 5 (A) shows the plasma concentrations, and Figure 5(B) shows the lung deposition amount after PRF administration in each group. In Figure 5 (A), comparing the SDH, SDM, and SDL groups, the SDM group received twice the amount of the SDL group, but C_max_ increased about 7-fold to 3.7 μg/mL and 21.8 μg/mL, respectively. The C_max_ of SDH rats, administered twice the amount as SDM rats, was 35.4 μg/mL; although C_max_ did not reach twice that of the SDM group, the AUC increased to about 2-times that of the SDM group.

Comparing the three different dose groups, the SDL dose may not be as bioavailable as the SDM dose because the SDL dose is too small to evenly distribute the particles in the lungs. In addition, compared with the SDM group, the C_max_ of the SDH group did not double, but the AUC doubled. Comparing the oral and inhaled groups, the SDL, SDM, and SDH groups had increased relative bioavailability due to the avoidance of the first-pass hepatic metabolism and the large surface area of the lung. In the SDM group administered half the dose, the C_max_ and AUC were approximately twice as high as those of the oral SDM group. Comparing the SDH and the oral groups to which the same dose was administered, the AUC of the SDH group was approximately four times higher. Similar results are shown in the lung distribution profile in Figure 5 (B). Comparing SDL, SDM, and SDH groups, the higher the dose, the higher the C_max_. In addition, when compared with the oral group, the SDM group administered half the dose but showed the same C_max_ as the oral group. Therefore, based on plasma levels and lung distribution, PRF is more bioavailable when administered through inhalation than oral administration.

### 3.4. In vivo PD studies

**Figure 6.**
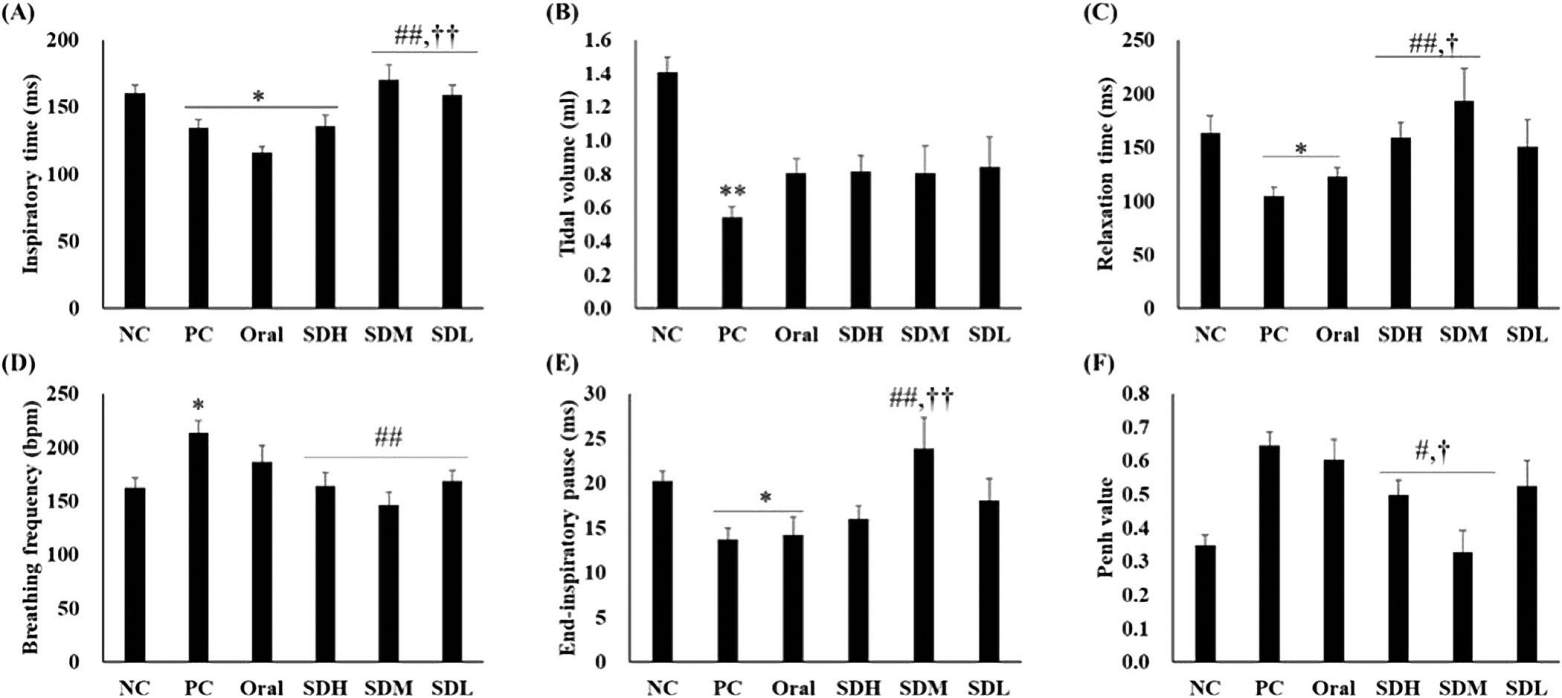
Lung function of bleomycin-induced idiopathic pulmonary fibrosis in rats after drug administration (*n* = 5, mean ± standard error). (A) Inspiratory time. (B) Total volume. (C) Relaxation time. (D) Breathing frequency. (E) End-inspiratory pause. (F) Penh-value. * ANOVA, *p*-value < 0.05 compared with NC; # ANOVA; ** ANOVA, *p*-value < 0.005 compared with NC; # ANOVA; *p*-value < 0.05 compared with PC; ## ANOVA; *p*-value < 0.005 compared with PC; † ANOVA, *p*-value < 0.05 compared with Oral; †† ANOVA, *p*-value < 0.005 compared with Oral.

Figure 6 shows the results of lung function assessment using DCP. Lung function decreased in the PC group in which IPF was induced by bleomycin. However, the lung function was improved the most in the PRF-treated group and was more effectively improved in the SD-PL 1:1-treated group (administration by instillation). All parameters, except tidal volume, were improved in the SDH, SDM, and SDL groups than in the PC group (Figure 6B). In particular, in Figure 6, for inspiratory time, relaxation time, end-inspiratory pause, and Penh-value, the lung function in the inhalation-administered group was improved compared to the oral administration group. Furthermore, lung function was effectively increased in the SDM group at the inspiratory time, relaxation time, and Penh-value compared with the oral group. This may indicate that IPF treatment was more effective due to improved bioavailability and transfer of a high amount of PRF to the target organ, the lung, as noted in the results of the PK studies.

**Figure 7.**
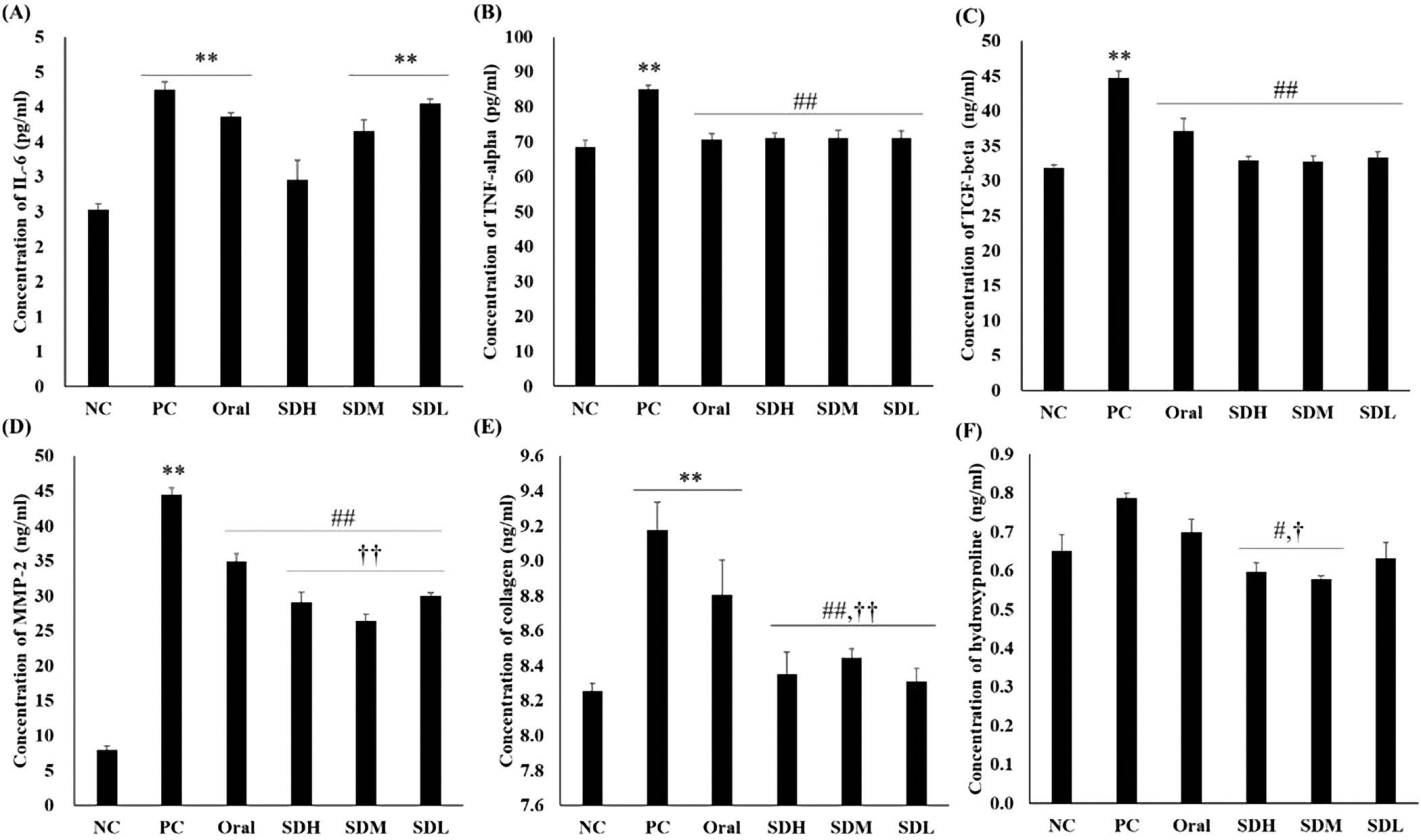
ELISA of bleomycin-induced idiopathic pulmonary fibrosis in rats after drug administration (*n* = 5, mean ± standard error). (A) Interleukin-6 (IL-6). (B) Tumor necrosis factor-alpha (TNF-α). (C) Transforming growth factor-beta (TGF-β). (D) Matrix metalloproteinase-2 (MMP-2). (E) Collagen. (F) Hydroxyproline. ** ANOVA, *p*-value < 0.005 compared with NC; # ANOVA, *p*-value < 0.05 compared with PC; ## ANOVA, *p*-value < 0.005 compared with PC; † ANOVA, *p*-value < 0.05 compared with Oral; †† ANOVA, *p*-value < 0.005 compared with Oral.

Figure 7 shows the quantitative results of cytokines and proteins involved in IPF using ELISA assays. The assays were performed with the BALF obtained after extracting the rat lungs. Cytokine and protein levels increased in the IPF-induced PC group compared to the NC group. As shown in Figure 7 (A), the levels of all proteins, except IL-6, decreased in the PRF-administered group compared with the PC group. The levels of MMP-2, hydroxyproline, and collagen, which participate in collagen synthesis, decreased in the inhaled groups compared to the oral group. Similarly, there was a significant decrease in the levels of these proteins in the SDM group administered half the dose compared with the oral group. In particular, there was a significant difference in MMP-2 and collagen levels between the SDM group administered the dose orally, and the SDM group administered a quarter of the dose. From the results of PK evaluation, it is considered that the suppression of cytokine expression by PRF is more effective because the inhalation-administered groups had high deposition amount of lung, with PRF showing drug effects at a higher concentration. As it has an excellent effect in suppressing the progression of IPF, collagen fibrosis did not progress significantly, and lung function was considerably improved.

**Figure 8.**
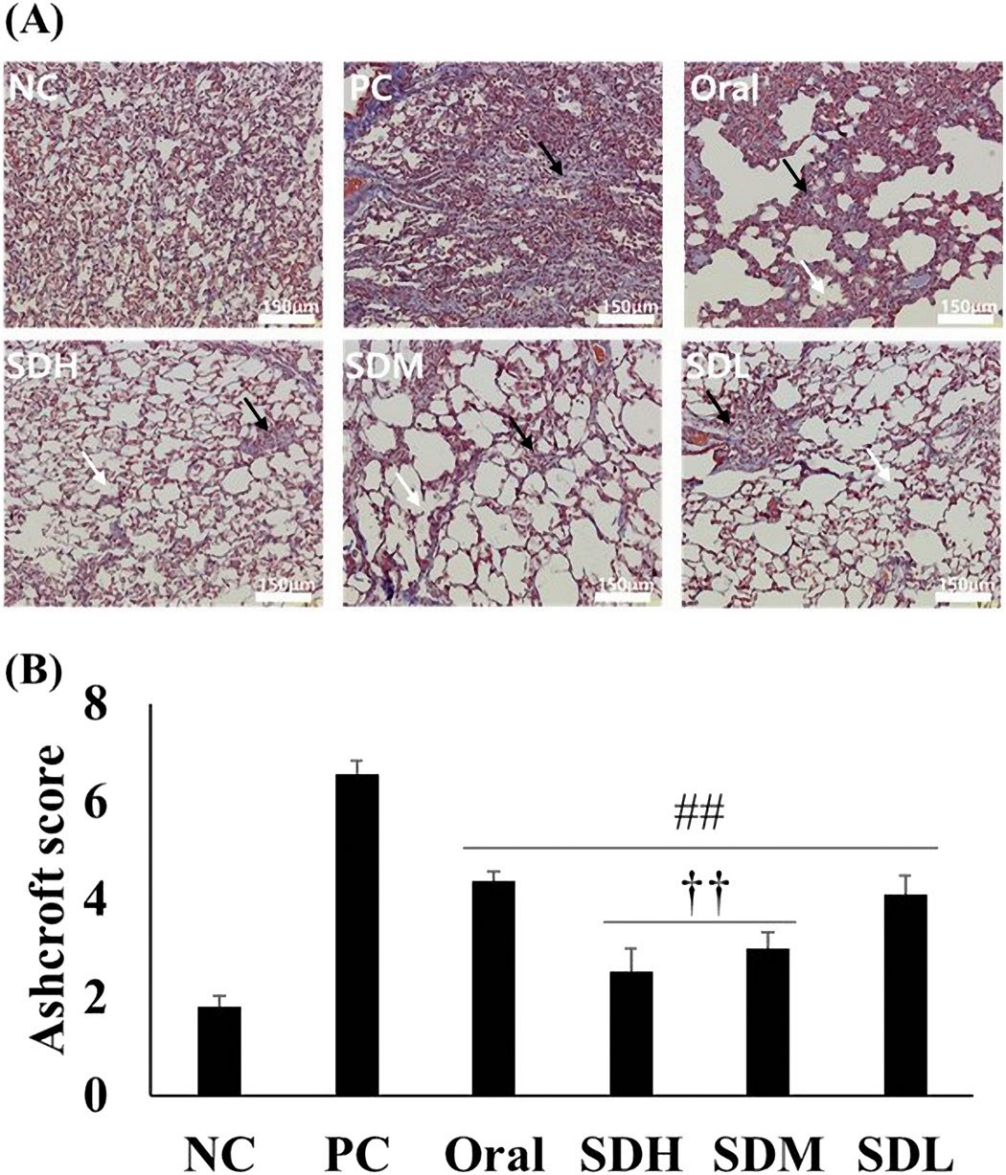
Histological evaluation of lungs with bleomycin-induced idiopathic pulmonary fibrosis in rats after drug administration (*n* = 5, mean ± standard error). (A) Microscopic image of lung section stained with trichrome. White arrow: broken alveolar wall; Black arrow: fibrosis mass (B) Ashcroft score of microscopic analysis. ## ANOVA, *p*-value < 0.005 compared with PC; †† ANOVA, *p*-value < 0.005 compared with Oral.

Figure 8 shows microscopic images of the lungs of rats in each group and the corresponding Ashcroft scores. In the IPF-induced PC group, the alveoli were filled with fibrosis, and only the mass form was present. In addition, collagen (stained blue with Masson’s trichrome) was present over a large area. It was determined that the degree of IPF was lower in the oral administration group compared to the PC group because the space was more visible, and the shape of some alveoli was preserved. The SDL, SDM, and SDH groups showed a morphology closer to the NC group than the PC group. The SDH group samples showed broken alveolar walls and very few masses. In the SDM group samples, the alveolar wall was cut, the alveoli appeared to be enlarged, and the remaining alveolar wall was thickened. The SDL group samples also exhibited a broken alveolar wall and mass, primarily due to fibrosis. These microscopic images were scored by the observer based on the Ashcroft score (Figure 8 (B)). The PC group had the highest score, which decreased significantly in all PRF-treated groups. In particular, the scores for SDH and SDM groups were significantly lower than that for the oral administration group. Based on the evaluation of lung function, cytokine levels, and pathology performed in the current study, it was concluded that pulmonary-administration of PRF is more effective for IPF treatment than oral administration, even if half the dose of PRF is administered via the inhalation route.

## 4. Conclusion

We administered PRF via the pulmonary route rather than orally to reduce the side effects and dose, ultimately improving the therapeutic effect. To efficiently transmit to the deep lungs using the pulmonary route, the particle size had to be reduced to approximately 5 μm. However, PRF is unsuitable for atomizing into small sizes, owing to its high crystallinity and cohesiveness. Therefore, to improve these properties, we aimed to create PRF microparticles via co-spray drying with mannitol, NaCl, and Leu as stabilizers. NaCl and Leu were selected as effective stabilizers for PRF through PSD and SEM images and co-spray-dried with the ratio of PRF and stabilizer. XPS confirmed that Leu was abundant on the surface of the microparticles. Therefore, it is considered that SD-PL, due to the surface properties of Leu, could be effectively made up of small particles. It was also confirmed that SD-PL particles have more advantageous characteristics for inhalation in BET and true density.

Through ACI, we confirmed that SD-PL 1:1 particles with a PRF: Leu ratio of 1:1 had the best inhalation efficiency; therefore, we proceeded with PK and PD studies using SD-PL 1:1. Through *in vivo* PK studies, the bioavailability of PRF through the pulmonary route was found to be four times higher than that in the oral group. The SDM group administered half the dose had twice the C_max_ and AUC values as the oral group. The amount distributed in the lung, the target organ of the PRF, and SDH was also four times higher than that of oral group. *In vivo* PD studies confirmed the therapeutic effect of PRF in IPF-induced models through ELISA, DCP, and histology. These results further validated the PK study results. Our findings showed that the therapeutic effect was enhanced when PRF was administered via the pulmonary route. Nonetheless, cytokines and lung function mediated a higher therapeutic effect in the inhaled group than in the oral group. In conclusion, compared to a large oral dose, administering a low dose of SDM to the lungs helps achieve the same lung concentration and superior therapeutic effect. Therefore, it can be considered that the side effects are reduced by inhalation. Additionally, DPI does not pass through the gastrointestinal tract due to being administered via the lungs. We confirmed an increase in the bioavailability in the previous PK studies, and it is possible to reduce stomach and liver exposure to PRF through low dose administration of the SD-PL 1:1. This can reduce the side effects on the gastrointestinal tract and the liver. Overall, in this study, we confirmed that administering PRF via the pulmonary route efficiently enhanced the bioavailability, lung distribution, and therapeutic effects of PRF in the treatment of IPF.
